# A Review: Expression of Aquaporins in Otitis Media

**DOI:** 10.3390/ijms18102164

**Published:** 2017-10-17

**Authors:** Su Young Jung, Sung Su Kim, Young Il Kim, Sang Hoon Kim, Seung Geun Yeo

**Affiliations:** 1 Department of Otorhinolaryngology-Head and Neck Surgery, Graduate School, Kyung Hee University, Seoul 130-701, Korea; monkiwh35@hanmail.net (S.Y.J.); hoon0700@naver.com (S.H.K.); 2 Department of Biochemistry and Molecular Biology, Medical Science and Engineering Research Center for Bioreaction to Reactive Oxygen Species, BK-21, School of Medicine, Kyung Hee University, Seoul 130-701, Korea; sgskim@khu.ac.kr; 3 Medical Science Research Institute, Kyung Hee University Medical Center, Seoul 02447, Korea; kimyi88@empas.com

**Keywords:** aquaporin, water channel, transmembrane permeability, human middle ear, otitis media

## Abstract

Otitis media (OM) refers to inflammatory diseases of the middle ear (ME), regardless of cause or pathological mechanism. Among the molecular biological studies assessing the pathology of OM are investigations of the expression of aquaporins (AQPs) in the ME and Eustachian tube (ET). To date, fifteen studies have evaluated AQPs expression in the ME and ET. Although the expression of individual AQPs varies by species and model, eleven types of AQP, AQP1 to AQP11, were found to be expressed in mammalian ME and ET. The review showed that: (1) various types of AQPs are expressed in the ME and ET; (2) AQP expression may vary by species; and (3) the distribution and levels of expression of AQPs may depend on the presence or absence of inflammation, with variations even in the same species and same tissue. Fluid accumulation in the ME and ET is a common pathological mechanism for all types of OM, causing edema in the tissue and inducing inflammation, thereby possibly involving various AQPs. The expression patterns of several AQPs, especially AQP1, 4 and 5, were found to be altered in response to inflammatory stimuli, including lipopolysaccharide (LPS), suggesting that AQPs may have immunological functions in OM.

## 1. Introduction

Otitis media (OM) refers to inflammatory diseases of the middle ear (ME), regardless of cause or pathological mechanism [1]. In children, OM is the leading reason for doctor visits, prescribing antibiotics and performance of surgical procedures, as well as being the most common cause of hearing loss [2,3,4]. In addition to being classified by cause, clinical course, and pathological mechanism, OM can be classified based on the presence or absence of middle ear effusion and the characteristics of the effusion. Effusion indicates fluid produced by infections or inflammatory responses in the mucosa, which can be caused anywhere in pneumatized temporal bone.

Three types of effusion have been identified: serous, mucoid and suppurative. In practice, OM can be generally categorized as OM with effusion (OME) and as suppurative OM (SOM). OME, also known as serous or secretory OM, is defined as OM accompanied by fluid accumulation in the ME, with no perforation of the tympanic membrane, whereas SOM is characterized by a purulent discharge with perforation of the tympanic membrane. Based on duration, OM can be divided into three types: acute OM, lasting <3 weeks; subacute OM, lasting ≥3 weeks but <3 months; and chronic OM, lasting >3 months. Based on its clinical manifestations, however, OM can be generally classified as acute OM (AOM), OM with effusion (OME), chronic OM (COM) and cholesteatomatous OM [5]. Moreover, OM can be classified by causes, which are usually complex and multifactorial. The most important contributing factors are Eustachian tube (ET) dysfunction and microbial infection [6]. Once ET dysfunction or microbial infection induces inflammation in the ME, the pattern and progression of OM will vary, depending on each individual’s conditions, immune responses and biochemical factors, which are thought to be related to the recurrence and chronic character of OM. Studies in animals and of the histopathology of the temporal bone in humans, designed to elucidate the pathogenesis of OM, have suggested that the histopathological, biochemical and immunological findings in different types of OM are closely correlated with each other, leading to transitions among these different types of OM [7,8,9,10].

Among the molecular biological studies assessing the pathology of OM are investigations of the expression of aquaporins (AQPs) in the ME cavity and ET. AQPs, also called water channels, are integral membrane proteins and members of a larger family of major intrinsic proteins that form pores in the membranes of biological cells, which facilitate the transport of water between cells [11]. All cells, including mucous cells, require water to survive and function physiologically. Water moves in a systemic way rather than simply through cell-to-cell contact. AQPs regulate one of the steps in water movement by selectively transporting water molecules into and out of cells, but preventing the movement of ions and other solutes [12]. The distribution of AQPs in the ear may be closely correlated with both the normal physiology and the pathophysiology regarding the movement of water, ions and chemicals in the ET and ME. This review of research on AQPs in the ET and ME was performed to (1) investigate the characteristics of AQP that play an important role in water homeostasis of the ME; and (2) determine whether AQP-associated changes in water homeostasis in the ME and ET could trigger OM.

## 2. General Characteristics of Aquaporins (AQPs)

### 2.1. Classification of AQPs in Mammals

**Figure 1:**
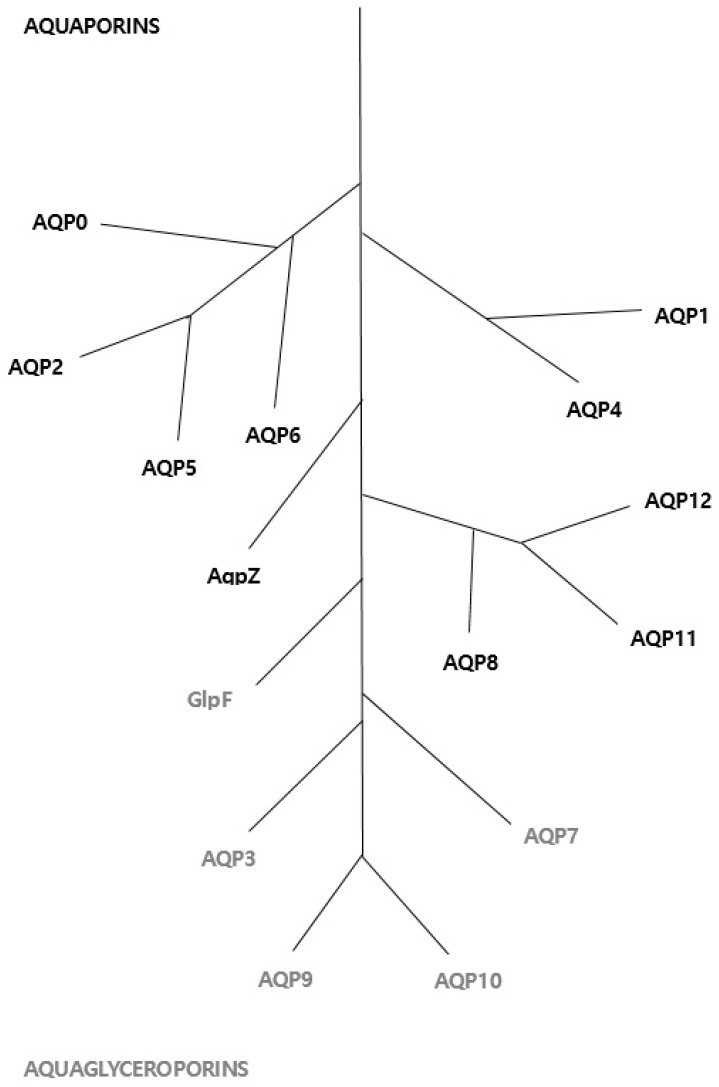
Phylogenetic leaf of the human aquaporin gene family. Water permeable aquaporins are shown in bold (AQP0, 1, 2, 4, 5, 6, 8, AqpZ). Glycerol permeable aquaglyceroporins are in italics (AQP3, 7, 9, 10, GlpF). *E. coli* homologues are AqpZ and GlpF. The unclassified subfamily comprising AQP11 and 12 is on the bottom right. The scale bar represents genetic distance between homologues.

To date, 13 AQPs have been identified, designated AQP0 to AQP12. These proteins have been divided into subgroups based on their structural and functional characteristics: (1) classical aquaporins (AQP0, AQP1, AQP2, AQP4, AQP5; known as orthodox AQPs) permeable only to water molecules; (2) aquaglyceroporins (AQP3, 7, 9 and 10) permeable to glycerol, urea and other small solutes in addition to water; (3) a third group that was recently isolated, the so-called unorthodox aquaporins (AQP11 and AQP12), which share low homology with other proteins from this family; and (4) AQP6 and AQP8, which have very recently been classified as unorthodox aquaporins [13,14,15] (Figure 1).

### 2.2. Function of AQPs

AQPs are intrinsic membrane proteins that passively transport water across cell membranes, while preventing the passage of ions and other solutes. AQPs are involved in the migration of water in various cells. Moreover, tumor induction in AQP1 knockout mice resulted in smaller and slower growing tumors than in wild-type mice [16]. In addition, AQP knockout slowed astrocyte migration in response to chemotactic stimulus, and delayed glial scar formation in recovering impaired brain tissue [17]. Also, the aquaglyceroporins (AQP3, 7, 9 and 10) were found to transport small uncharged solutes, such as glycerol, CO_2_, ammonia and urea, depending on pore size [18]. This was verified by results showing reduced stratum corneum hydration in AQP3 knockout mice [19] and fat accumulation or obesity in AQP7 knockout mice [20]. Taken together, these results suggest that the aquaglyceroporins, which transport water as well as glycerol, are associated with lipid metabolism by regulating glycerol levels in the epidermis. In addition, neurons as well as glial and Müller cells, which protect bipolar cells in the retina, are positive for AQPs and respond to a signal from neurons via AQP4. The reduced sensitivity to light and sound observed in AQP4 knockout mice strongly supports the hypothesis that AQPs may be involved in neuronal signal transduction [21].

### 2.3. Regulation of AQP Expression

Expression of AQPs is regulated by external stimuli and/or alterations in environmental conditions, with the regulatory mechanism classified as short- or long-term. A representative short-term regulatory mechanism is phosphorylation. Binding of vasopressin to its receptor activates protein kinase A (PKA), which phosphorylates the Ser256 residue of AQP2 and increases water migration through AQP2 [22,23]. Heavy metals are also involved in the short-term regulation of AQP expression, with mercury inactivating most AQPs, except for AQP4 [24], and nickel and copper inactivating AQP3, thereby increasing the permeability of AQP4 [25]. Furthermore, pH change and copper alter the expression of AQP3 [26].

Long-term regulation can be divided into regulation during the development stage and regulation through interaction between proteins. AQP4 is first expressed two weeks after birth, with its level gradually increasing thereafter. Moreover, its expression patterns vary, depending on location and timing, with transcription of the two isoforms regulated differently. In addition, the expression of AQPs is controlled by protein–protein interactions, including interactions with dystrophin and syntrophin. For example, the dystrophin-deficient *mdx* mouse, an animal model of Duchenne muscular dystrophy (DMD), showed reduced AQP4 expression in muscle cells, indicating that AQP4 interacts with the dystrophin-associated complex DAP [27,28]. Evaluation of α-syntrophin knockout mice showed that, although the overall expression of AQPs was unaltered compared with wild-type mice, the expression of AQPs in perivascular and subpial cellular membranes was lower in knockout mice. In addition, ubiquitination was found to regulate AQP1 expression [29]; steroid hormones and osmotic pressure can control the expression of AQP1, 4 and 9; and oxygen level has been reported to alter expression of some AQPs [30].

## 3. Types and Functions of AQPs Expressed in the Middle Ear and Eustachian Tubes

### 3.1. Expression of AQPs in the Middle Ear and Eustachian Tubes

To date, fifteen studies have evaluated AQPs expression in the ME and ET (Table 1) [31,32,33,34,35,36,37,38,39,40,41,42,43,44,45]. These include six studies in rat models, two in mouse models, three in guinea pig models, and three in human patients. AQPs expression was evaluated by immunohistochemistry, real-time polymerase chain reaction (qPCR) and Western blotting in four, twelve and five studies, respectively. Although the expression of individual AQPs varies by species and model, eleven types of AQP, AQP1 to AQP11, were found to be expressed in mammalian ME and ET, with Western blotting or immunohistochemistry (IHC) confirming the expression of four AQPs proteins; i.e., AQP1, 3, 4 and 5. These results, showing that various types of AQPs are expressed in ME and ET, suggest that AQPs may play various roles in the pathophysiology of OM.

### 3.2. Pathophysiologic Roles of AQPs Expressed in the Middle Ear and Eustachian Tubes

#### 3.2.1. Aquaporin 1 (AQP1)

AQP1 is a protein encoded in humans by the *AQP1* gene. Although distributed in various tissues, its physiological activity is especially pronounced in the kidneys, where it serves as a water channel. AQP1 is expressed in the basolateral and apical membranes of the proximal tubules, the descending limb of the loop of Henle, and the descending portion of the vasa recta. Additionally, this protein is found in red blood cells, vascular endothelium, sweat glands, gastrointestinal tract, and lungs. AQP1 may also be involved in disorders involving imbalances in ocular fluid movement [45]. In the ME and ET, AQP1 is distributed on the surfaces of capillary endothelial cells and subepithelial fibroblasts, which are separated from the ME by the basement membrane [33]. This finding was consistent with results showing the presence of AQP1 in capillaries of various tissues, indicating that AQP1 regulates water transport between blood and feeder cells of these tissues [46]. In addition, immunomorphologic studies confirmed that AQP1 is expressed in fibroblasts of the ME [39] and inner ear [47], and that these fibroblasts maintain fluid transport and ion gradients. Taken together, these results indicate that AQP1 may be involved in water homeostasis in the subepithelial milieu of the ME.

#### 3.2.2. Aquaporin 2 (AQP2)

AQP2 is found in the apical cell membranes of the principal cells in the collecting ducts of kidneys and in their intracellular vesicles. The primary function of AQP2 is water reabsorption from urine and returning the reabsorbed water into the blood stream. AQP2 is present in kidney epithelial cells and usually lies dormant in intracellular vesicle membranes. When cells require AQP2, vasopressin binds to the cell surface vasopressin receptor, activating a signaling pathway that causes the AQP2-containing vesicles to fuse with the plasma membrane [48]. The microstructure and functions of the inner ear and kidneys are similar. Because AQP2 is also expressed in the endolymphatic sac, the main organ regulating endolymph, and AQP2 expression is regulated by arginine vasopressin (AVP) infusion, the AVP-AQP2 system may be involved in regulating endolymph in the inner ear [49].

No study to date has assessed AQP2 expression in the human ME or ET. However, analysis of uninfected and infected ME tissue of the C3H/HeJ mouse model of COM found that AQP2 expression was higher, by 3.22 ± 0.47-fold and 2.79 ± 0.57-fold, respectively, than in ME tissue of control BLAB/c mice [36], suggesting that AQP2 upregulation in ME tissue may be unrelated to infection. Moreover, AQP2 gene expression was not significantly altered by the presence of bacteria or phosphate-buffered saline (PBS) stimulation [35]. Further studies are needed to elucidate the physiological and/or pathological roles of AQP2 in the ME or ET.

#### 3.2.3. Aquaporin 3 (AQP3)

AQP3 is found in the skin, lungs, cornea, esophagus, colon, stomach, liver, intervertebral discs and sperm. In addition, it is found in the basolateral cell membranes of principal collecting duct cells and provides a pathway for water to exit these cells [50]. IHC showed AQP3 expression on the basolateral membranes of ciliated epithelial cells of normal human middle ear epithelium (NHMEE) [33]. The embryological origin of ME epithelium is identical to that of respiratory epithelium. The cells of the alveolar epithelium (mainly type I epithelial cells) express AQP5 on their apical membranes and AQP3 on their basolateral membranes [51]. The location of AQP3 suggests that this protein may be involved in the movement of water between subepithelial connective tissues and epithelial cells [33]. In addition, AQP3 appears to control transepithelial water movement and the volume of water in the ME and ET [52].

A study of ME effusion in children who were diagnosed with OME and underwent ventilation tube insertion showed that the presence or absence of bacteria in ME effusion did not alter the expression of AQP3 [32]. In addition, AQP3 expression was not altered by the presence or absence of sinusitis or allergic rhinitis, the number of OME recurrences, or the number of previous ventilation tube insertions [32]. In mice, however, AQP3 expression was significantly upregulated by inflammation, reaching levels 5–6 times normal by 72 h and still increased at 1 week. AQP3 expression was not significantly altered at 6 hours, became moderately elevated (3-fold) at 24 h, and was highest at 3 days, suggesting that AQP3 may be involved in late stages of inflammatory responses. Although AQP3 expression is upregulated in response to both bacterial infection model and inoculation with PBS, the presence of bacteria seemed to delay AQP3 upregulation for 24 h. This finding suggests that AQP3 may be downregulated in response to inflammation (early response), but upregulated in response to fluid accumulation (late response) [35].

#### 3.2.4. Aquaporin 4 (AQP4)

AQP4 belongs to the AQP family of integral membrane proteins, which conduct water through cell membranes. AQP4 is the most prevalent aquaporin channel in the central nervous system (CNS), specifically located at the perimicrovessel astrocyte foot processes, glia limitans and ependyma [53]. Additionally, AQP4 is expressed on epithelial cells of many organs throughout the human body, including the kidneys, salivary glands, intestines, sensory organs and skeletal muscles [54], where it is concentrated within the basolateral membrane layers [55]. Furthermore, AQP4 plays a role in the supportive cells of sensory organs, such as the retina, inner ear, and olfactory epithelium [56]. Overall, AQP4 provides fast water transportation as well as being the primary water channel protein that maintains homeostatic balance within the CNS [57]. AQP4 may also be involved in various physiological processes, such as waste removal and fine-tuning of potassium homeostasis [58]. Within the inner ear, its main role is to provide osmotic balance in supporting epithelium cells within the organ of Corti by recycling K+ [54].

Although AQP4 mRNA was expressed in NHMEE, AQP4 protein was not detected by IHC. In the ME and ET of both rat [37,40,41] and guinea pig [38] models, however, AQP4 protein was detected by IHC and Western blotting, and AQP4 mRNA was expressed. These findings suggest that AQP4 expression may be species-dependent. Alternatively, AQP4 expression may be associated with the pathology of ME and ET inflammation. Because NHMEE consists of normal cells, AQP4 may not be expressed in these samples. In contrast, cells in the animal models were obtained following inflammation, as in OM. A study of normal ME tissue of mice showed the presence of anti-AQP antibody in ME epithelium, cytoplasm, and bones of ossicles. In contrast, a study of inflamed ME tissue of these mice showed the presence of anti-AQP antibody in the ME epithelium, cytoplasm and mucosal layer of the tympanic membrane, as well as in inclusion bodies and epithelium on the lateral ME surface of round window membranes. Taken together, these findings indicate that AQP4 expression in the ME may be regulated by the condition of the ME [59].

Injection of lipopolysaccharides (LPS), an endotoxin of Gram-negative bacteria that induces innate immune responses, into the ME and ET showed greater enhancement of APQ4 in ME than in the ET. This may occur because immune responses against antigens are highly activated, and lymphocytes can more easily move to mucous membranes of normal ME, although these mucous membranes have no lymph tissue and few leukocytes [38,39,60].

#### 3.2.5. Aquaporin 5 (AQP5)

AQP5 is a protein encoded in humans by the *AQP5* gene. AQP5 plays a role in the generation of saliva, tears and pulmonary secretions [61]. Pilocarpine-stimulated fluid secretion was reduced more than twofold in AQP5 knockout mice. AQP5 thus facilitates fluid secretion in submucosal glands, indicating that the luminal membrane of serous epithelial cells is the rate-limiting barrier to water movement [62].

A study using NHMEE showed that AQP5 was expressed on the apical surface of epithelial stratified cells [33]. AQP5 on the luminal surface of epithelium may enable water to move between the epithelial cells and the cavity of the ME. AQP5 also appears to be able to control transepithelial water movement and the volume of water in the ME [52]. In lung tissue, AQP5 expression increases in response to stimuli such as tumor necrosis factor (TNF) and LPS, showing a similar pattern in patients with OME [31,38]. The important roles of AQP5 in innate immune responses and maintaining epithelial barrier integrity suggest that AQP5 can regulate mucus secretion. AQP5 promotes airway epithelial expression of an adhesion molecule (intercellular adhesion molecule) and the secretion of a chemokine (lipopolysaccharide-induced CXC) essential for neutrophil recruitment [63]. AQP5 also directly interacts with microtubules in epithelial cells, influencing paracellular permeability independent of water transport [64]. AQP5 mRNA was detected by RT-PCR in both the ME and ET of rats, whereas Western blotting with anti-AQP antibody showed the presence of AQP5 protein in the ET, but not in the ME. IHC showed AQP5 expression in the apical portion of serous gland cells of the ME and ET [40]. These findings suggest that AQP5 may be involved in the secretion or transport of water-rich (serous) fluid in serous gland cells [65]. Injection of LPS into the ME and ET of rats showed that AQP5 was expressed more rapidly and distinctively in the ET than in the ME, in contrast to the distribution of AQP4, a difference that may result from the presence of more serous gland cells in the ET than in the ME [44].

#### 3.2.6. Aquaporin 6–11 (AQP6–11)

Although AQP6 expression is specific to the kidneys, AQP6 has been detected in the retina, ovaries, parotid glands, and cochlear sensory epithelium [66]. Few studies to date have assayed AQP6 expression in the ME or ET. Although AQP6 mRNA was detected by RT-PCR in NHMEE, AQP6 protein was not detected by IHC in the same samples [33].

The sequence of AQP7 is closer to that of AQP3 and AQP9 than to other AQPs, suggesting that AQP3, 7 and 9 may constitute a subfamily. AQP7 facilitates water, glycerol and urea transport and may play an important role in sperm function [67]. Although few studies have assessed AQP7 expression in ME and ET, one study reported that AQP7 mRNA was expressed in rat ET [41]. In contrast, studies in rats [42] and NHMEE [13] found that AQP7 was not expressed at either the gene or protein level.

AQP8, encoded in humans by the *AQP8* gene, has shown a preference for neutral NH_3_ molecules over water, suggesting that this protein plays a physiological role in the maintenance of acid-base equilibrium. At physiological concentrations, AQP8 may augment basal NH_3_ conductivity 3- to 5-fold [68]. In humans, AQP8 mRNA was expressed only in the pancreas and colon [68]. In contrast, AQP8 mRNA was found to be expressed in NHMEE [33] and rat ET [41].

AQP9, a protein encoded in humans by the *AQP9* gene, allows passage of a wide variety of noncharged solutes. AQP9 stimulates urea transport and osmotic water permeability; there are conflicting reports about its role in providing glycerol permeability. AQP9 may have specialized functions in leukocytes such as in immune responses or bactericidal activity [69]. AQP9 expression has also been detected in human adipocytes and shown to function in transmembrane glycerol movement [70]. However, AQP9 mRNA expression has been detected only in rat ET [41].

AQP10 is an aquaglyceroporin expressed only in human small intestine, in contrast to mice, where it was shown to be a pseudogene [71]. AQP10 gene expression has been reported in NHMEE [33]. Moreover, the pattern of AQP10 gene expression was shown to be similar to that of CXCL4 expression in children with OME [32]. However, AQP10 gene expression in ME effusion fluid was found not to depend on the presence or absence of bacteria or comorbidities (e.g., allergic rhinitis or sinusitis) or the numbers of OME recurrences or previous ventilation tube insertions. AQP10 plays a key role in the pathogenesis of pompholyx, including its involvement in blister formation, inflammation and drying, but its exact pathophysiological function in ME and ET has not yet been identified.

AQP11 is a protein with a lower degree of homology to previously characterized aquaporins and aquaglyceroporins. AQP11 was previously described in mice [72], although solute transport could not be measured. AQP11 RNA and protein has been detected in multiple rat tissues, including kidney, liver, testes and brain [73]. RT-PCR showed that AQP11 mRNA was expressed in NHMEE samples from ME and ET; however, IHC failed to detect AQP11 protein in these samples.

Despite various studies assessing the tissue distribution and pathophysiological function of AQP6-11, to our knowledge no study has focused on their distribution or function in ME and ET. Additional studies are needed to investigate the function of each AQP subtype.

### 3.3. Summary of the Role of Presumed AQPs in the Pathogenesis of Otitis Media

To date, 13 AQPs have been found in mammals, designated AQPs 0–12. Nine of these, AQPs 1–6, 8, 10 and 11, have been identified in human or animal middle ears [31,32,33,34,35,36,37,38,39,40,41,42,43,44,45]. Previous studies on the human middle ear found AQP 5 in the middle ear mucosa, AQPs 3 and 10 in the effusion of OME, and AQPs 1, 2, 3, 4, 5, 6, 8, 10 and 11 in normal human middle ear mucosa. However, few studies have examined the role and function of AQPs in acute otitis media, otitis media, chronic otitis media, and cholesteatomatous otitis media.

Although the accumulation of ME fluid is multifactorial, transepithelial water transport mechanisms are affected by bacterial inoculation [32,35,37] and may play a significant role in the development and persistence of ME effusion. In order for the mucosal epithelium of the ME and the Eustachian tubes to maintain a normal defense mechanism, it is necessary to maintain the homeostasis of the mucous layer and the periciliary fluid layers produced by ciliated cells and goblet cells and to control the amount of water at appropriate levels [11,12].

Changes in water and electrolyte components in the middle ear mucous membrane may be a cause of otorrhea and presence of effusion in the middle ear, and may give rise to middle ear diseases such as OM and cause them to become chronic conditions [11,13,15]. It is assumed that aquaporins are one of the factors involved in the regulation of water.

## 4. Conclusions

This review has summarized current knowledge about the distribution and function of AQPs in ME and ET, and their associations with mechanisms that may influence the pathophysiology of OM. The review showed that (1) various types of AQPs are expressed in the ME and ET; (2) AQP expression may vary by species; and (3) the distribution and levels of expression of AQPs may depend on the presence or absence of inflammation, with variations even in the same species and same tissue. Fluid accumulation in the ME and ET is a common pathological mechanism for all types of OM, causing edema in the tissue and inducing inflammation, thereby possibly involving various AQPs. The expression patterns of several AQPs, especially AQP1, 4 and 5, were found to be altered in response to inflammatory stimuli, including LPS, suggesting that AQPs may have immunological functions in OM. Furthermore, the involvement of AQPs in neuronal signal transduction as well as regulating cell movement and lipid metabolism suggests that these findings may be used to develop new treatments for OM of the ME and ET. Additional studies are required to determine the expression and function of AQPs in the physiological and pathological states of the ME and ET, as well as to identify the factors that interact with AQPs and to determine how AQPs are modulated by these factors.

## Figures and Tables

**Table 1:** The studies of expression of AQPs in middle ear and eustachian tube.

Authours and References	Aquaporins	Species and/or Tissue Type					Detection Method		
		Rat or Mice	Guinea Pig	Human			IHC	qPCR	Western Blotting
				NHMEE	OME-M	OME-E			
Samuel et al. [31]	5				●			●	
Jin et al. [32]	3,10					●		●	
Seo et al. [33]	1,2,3,4,5,6,8,10,11 1,3,5			● ●			●	●	
Yu et al. [34]	1		●				●	●	●
MacArthur et al. [35]	1,2,3,5	●						●	
MacArthur et al. [36]	1,2,3,5	●						●	
Song et al. [37]	1,4,5	●					●	●	
Zhang et al. [38]	4,5		●					●	●
Zhang et al. [39]	1		●					●	●
Kang et al. [40]	1,4,5	●						●	●
Chun et al. [41]	1,3,4,5,7,8,9	●						●	
Chang et al. [42]	1,9	●					●		
JIn et al. [43]	1,4,5 1,4	● ●						●	●
Ahn et al. [44]	4,5	●							
Morris et al. [45]	1,4,5	●					●		

NHMEE: normal human middle ear epithelium; OME-M: mucosa of middle ear cavity from patients with otitis media with effusion; OME-E: effusion of middle ear cavity from patients with otitis media with effusion; IHC: immunohistochemistry; qPCR: real-time polymerase chain reaction.
